# Parkinson's disease and Parkinsonism syndromes: Evaluating iron deposition in the putamen using magnetic susceptibility MRI techniques - A systematic review and literature analysis

**DOI:** 10.1016/j.heliyon.2024.e27950

**Published:** 2024-03-26

**Authors:** Sana Mohammadi, Sadegh Ghaderi

**Affiliations:** a Department of Medical Sciences, School of Medicine, Iran University of Medical Sciences, Tehran, Iran; b Department of Neuroscience and Addiction Studies, School of Advanced Technologies in Medicine, Tehran University of Medical Sciences, Tehran, Iran

**Keywords:** QSM, SWI, Putamen, Iron, Parkinson's disease, Parkinsonism

## Abstract

Magnetic resonance imaging (MRI) techniques, such as quantitative susceptibility mapping (QSM) and susceptibility-weighted imaging (SWI), can detect iron deposition in the brain. Iron accumulation in the putamen (PUT) can contribute to the pathogenesis of Parkinson's disease (PD) and atypical Parkinsonian disorders. This systematic review aimed to synthesize evidence on iron deposition in the PUT assessed by MRI susceptibility techniques in PD and Parkinsonism syndromes. The PubMed and Scopus databases were searched for relevant studies. Thirty-four studies from January 2007 to October 2023 that used QSM, SWI, or other MRI susceptibility methods to measure putaminal iron in PD, progressive supranuclear palsy (PSP), multiple system atrophy (MSA), and healthy controls (HCs) were included. Most studies have found increased putaminal iron levels in PD patients versus HCs based on higher quantitative susceptibility. Putaminal iron accumulation correlates with worse motor scores and cognitive decline in patients with PD. Evidence regarding differences in susceptibility between PD and atypical Parkinsonism is emerging, with several studies showing greater putaminal iron deposition in PSP and MSA than in PD patients. Alterations in putaminal iron levels help to distinguish these disorders from PD. Increased putaminal iron levels appear to be associated with increased disease severity and progression. Thus, magnetic susceptibility MRI techniques can detect abnormal iron accumulation in the PUT of patients with Parkinsonism. Moreover, quantifying putaminal susceptibility may serve as an MRI biomarker to monitor motor and cognitive changes in PD and aid in the differential diagnosis of Parkinsonian disorders.

## Highlights

• QSM and SWI detect abnormal iron accumulation in the putamen of Parkinsonism.
• Comparative analysis unveils iron differences in PD vs. atypical Parkinsonism.
• Increased putaminal iron in PD linked to disease severity and cognitive decline.

## 1 Introduction

Magnetic resonance imaging (MRI) is a widely available and powerful tool for better neuroimaging visualization and has become an indispensable tool for evaluating and diagnosing the progression of neurodegenerative diseases (NDDs) [1,2]. Magnetic susceptibility (χ) is a property of a material that reflects its response to an external magnetic field [3,4]. Iron content, tissue microstructure and composition, and other factors have an impact on χ [[5], [6], [7]].

Iron is an essential element that plays a vital role in many biological processes in a variety of cellular processes, including oxygen transport, energy metabolism, and neurotransmitter synthesis [8,9]. Iron deposition has been implicated in the pathogenesis of these diseases, and MRI techniques are increasingly being used to quantify iron levels in the brain [10,11]. Therefore, MRI susceptibility methods can potentially detect and quantify iron and neurodegeneration in the brain [8,12]. These methods include T2*-weighted (T2*-w) imaging, susceptibility-weighted imaging (SWI), quantitative susceptibility mapping (QSM), R2* mapping, and phase imaging [[13], [14], [15]]. These techniques are sensitive to iron accumulation in the brain and can be used to monitor the progression of diseases with neurodegeneration [16] such as multiple sclerosis (MS) [17], Parkinson's disease (PD) [18,19], Alzheimer's disease (AD) [[20], [21], [22]], Huntington's disease (HD) [23], amyotrophic lateral sclerosis [24], and Wilson's disease [25].

Parkinsonism syndromes are a group of NDDs that include combinations of motor problems such as bradykinesia, resting tremor, rigidity, flexed posture, freezing, loss of postural reflexes, and non-motor features [[26], [27], [28], [29]]. PD is the major cause of Parkinsonism and the second most common NDDs. It is a slowly progressive disease characterized by the loss of neuromelanin-containing monoamine neurons, particularly dopamine neurons in the substantia nigra (SN) pars compacta [[30], [31], [32], [33], [34]]. Idiopathic PD (iPD) is a typical progressive neurodegenerative condition that affects approximately 1% of adults over the age of 65 [27]. The following conditions can mimic iPD called atypical Parkinsonism: multiple system atrophy (MSA), corticobasal degeneration (CBD), progressive supranuclear palsy (PSP), and dementia with Lewy bodies (DLB) [29,[35], [36], [37]]. Other causes of Parkinsonism include secondary causes, such as vascular Parkinsonism (VaP) and drug-induced Parkinsonism, genetic causes, tremor disorders, and non-neurological differentials of PD [29,38].

Conventionally, the basal ganglia, including the putamen (PUT), appear markedly hypointense on T2*-magnitude images due to high iron concentrations [[39], [40], [41]]. R2* relaxometry measures the reversible dephasing of proton spin echoes in the presence of field inhomogeneities and provides a quantitative index of iron deposition [42]. SWI combines both magnitude and phase information to enhance the contrast of tissues with different magnetic susceptibilities, such as iron-rich regions [43]. SWI exhibits sensitivity towards substances that distort the nearby magnetic field, such as iron [43,44]. In this scenario, phase information can be utilized to discern the differences [44]. Advanced MRI techniques, such as QSM, quantitatively measure bulk tissue χ, which strongly depends on iron content [45]. Local magnetic field inhomogeneities caused by iron can be probed using MRI phase and susceptibility mapping techniques [46]. These susceptibility methods can also enable visualization of the PUT structure and increase iron deposition during Parkinsonian disorders.

The structure and volume of the PUT can be altered in various neurological and neuropsychiatric conditions [47,48], including PD [48], AD [49,50], amyotrophic lateral sclerosis (ALS) [51,52], obstructive sleep apnea (OSA) [53,54], and major depressive disorder (MDD) [55]. Parkinsonian Syndromes are notably associated with elevated iron concentration in the deep gray matter (DGM) nuclei [[56], [57], [58], [59]]. It has been suggested that the PUT is one of the primary regions affected by Parkinson's syndrome [[60], [61], [62], [63]], which is age-dependent [48,59,64] and most prominently susceptible to the accumulation of iron [59,64]. Several studies, including meta-analyses, have also highlighted the significant accumulation of iron in the PUT of patients with Parkinsonism syndromes and PD, using postmortem and MRI measurements, including conventional MRI, SWI, and QSM techniques [57,65]. Iron deposition patterns have been observed in early- and middle-late-onset PD, emphasizing alterations in the iron levels in PUT [[66], [67], [68]]. The association between iron and PUT emphasizes the importance of iron metabolism in Parkinsonian syndromes.

Presently, existing literature contains several studies exploring iron accumulation in regions such as the PUT, SN, red nucleus (RN), and globus pallidus (GP) in various diseases [10,64,67,69]. However, there is a notable absence of comprehensive review articles addressing the specific topic of iron accumulation and deposition in PUT within these disorders. Given that PUT plays a pivotal role in the basal ganglia circuitry, impacting both motor and cognitive functions, it is particularly susceptible to irregular iron regulation and associated neurodegenerative processes [3,70,71]. Keeping a close watch on iron levels and neurodegenerative alterations in PUT holds significant importance for gaining insights into the underlying pathophysiology and progression of these conditions [72,73]. Additionally, this monitoring could aid in the development of potential biomarkers and therapeutic strategies [72].

In the past decade, advanced and novel MR susceptibility measurement techniques, such as QSM and SWI, have been commonly used in iron research [10,13]. The purpose of this article is to systematically review the literature on the use of χ MRI techniques in the assessment of iron deposition in Parkinsonism syndromes.

## 2 Materials and methods

### 2.1 Search strategy/inclusion and exclusion criteria

A comprehensive literature search followed the Preferred Reporting Items for Systematic Reviews and Meta-Analyses (PRISMA) statement [74]. The systematic review was not registered in a publicly accessible database, such as PROSPERO. PubMed and Scopus databases were used. The search strategy used a combination of keywords and terms to retrieve the relevant articles. Search syntax was tailored to each database to ensure that the results were accurate. (Supplementary Table 1). We also checked the reference lists of relevant articles to identify additional studies.

The eligibility of the studies for review was assessed using the following criteria: to be included, the studies had to use MRI to measure χ in the PUT (including QSM, SWI, T2*, and R2* mapping), focus on PD and other Parkinsonism syndromes including MSA, PSP, CBD, DLB, report imaging data on χ in the PUT, compare χ in the PUT between different groups of subjects (e.g., patients with neurodegenerative diseases and healthy controls (HCs)), and be published in English. In this study, we excluded records that did not meet these criteria and did not involve humans (e.g., animal or in vitro studies), reviews, case reports, editorials, letters, comments, or expert opinions. Two reviewers independently screened the titles and abstracts of the retrieved records and assessed full-text articles for eligibility.

### 2.2 Data extraction and quality assessments

The process of selecting the studies for this review involved multiple steps. The titles and abstracts of these studies were screened to assess their relevance. After this initial screening, full-text articles were obtained for potentially relevant studies based on the title and abstract. The full-text articles were evaluated in depth using predefined inclusion and exclusion criteria. Two independent reviewers extracted data from the included studies using a standardized data collection form. The extracted data included details about the study, such as the MRI device used, technique for measuring iron content, sample size, and key findings related to iron accumulation in the PUT for Parkinsonism syndromes. Any disagreements between the two reviewers during data extraction were resolved through discussion, and consensus was reached. We evaluated the quality of the included studies using the Newcastle-Ottawa Scale [75,76] for observational studies and Cochrane risk of bias tool for randomized controlled trials.

## 3 Results

### 3.1 Overview of the results

**Fig. 1:**
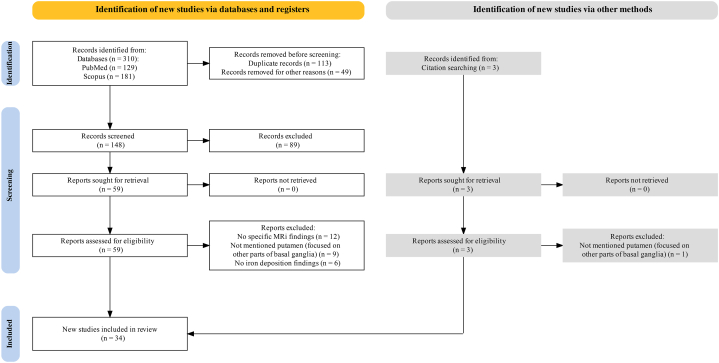
PRISMA flow diagram used to select articles for the systematic review analysis.

The initial search yielded 181 records from Scopus and 129 from PubMed. After screening titles, abstracts, and full-text articles for eligibility, as well as assessing citations from the included articles, 34 records were included in the systematic review (Table 1). A flow diagram of the study selection process is shown in Fig. 1. The MRI field strength varied from 1 T to 7T, with most studies using 3T scanners. Coil channels varied in studies, ranging from 8 to 32, with only one study using 64. The two most popular methods for χ measurement are QSM and SWI, with QSM being more commonly used in recent years.

**Table 1:** Summarizes putamen magnetic susceptibility MRI findings in Parkinson's disease and Parkinsonism syndromes.

Study	Field strength/Coil channels	Technique(s) for χ	Sample size	Main findings
Hanssen et al. (2023) [77]	1.5 T/20	SWI	Non-manifesting carriers: 10/XDP: 17/HC: 24	• XDP → ↑ χ^a^ than the HC in the medial PUT and the external pallidum bilaterally. • Iron deposition → anteromedial PUT (degenerative process starts in the anterior and medial parts).
Pang et al. (2022) [78]	3 T/32	SWI	iPD: 77/MSA-P: 75	• Iron accumulation of the dorsolateral PUT → more complex in MSA-P compared with iPD.
Mazzucchi et al. (2022) [79]	3 T/NR	QSM	PD: 31/MSA-c: 9/MSA-P:5/PSP: 24	• ↑ χ of the PUT → Optimal diagnostic accuracy in PD/MSA-p comparison. • ↑ χ of the PUT → Effective in distinguishing PD from PSP with good accuracy, though some overlap between the two groups exists.
Kang et al. (2022) [80]	3 T/24	QSM	PD: 104/HC: 45	• QSM signal intensity within PUT: ↑ in PD than HC. • ↑ Radiomics features from PUT → ↑ Correlations with MoCA scores in PD → Potential utility in evaluating cognitive impairment in PD. • ↔ Correlations between radiomics features and MoCA scores observed specifically in the neostriatum system (HCN and PUT), which are integral to the frontal lobe-striatal circuit implicated in cognitive impairment.
Zang et al. (2022) [81]	3 T/24	R2^a^ and SWI	PD: 28/HC: 25 fMRI: PD: 34/HC: 25 PET/fMRI: PD: 33/HC: 25	• Identified significant interaction effect of nigral iron deposition and nigral-PUT connectivity.
Du et al. (2022) [82]	3 T/NR	T2^a^ and QSM	PD drug-naïve: 18/PD drug-treated: 87/HC: 79	• Iron levels in the PUT did not differ between PD drug-naive and HC.
Prasuhn et al. (2022) [83]	3 T/NR	SWI	iPD: 30	• ↑ Subcortical brain iron deposition (PUT and GP) → ↑ Region-specific high-energy-containing phosphorus metabolites.
Zhao et al. (2022) [84]	3 T/16	QSM	PD-MCI: 16/PD-NC: 16/HC: 28	• ↑ χ in bilateral PUT distinguished PD-MCI from PD-NC. • ↑ χ in PD-MCI, no significant difference between PD-NC and HC. • Radiomic analysis revealed significant variance in bilateral PUT among three groups. • ↑ χ in PUT → ↑ motor deficit severity. • ↑ χ in PUT → ↓ MoCA-B scores. • ↑ PUT → ↑ UPDRS-III scores
Lancione et al. (2022) [85]	7 T/32	T2^a^, SWAN, and QSM	MSA-P: 19/MSA-c: 13/HC: 16	• ↑ Alterations in χ distribution observed in PUT for MSA-P.
Kim et al. (2021) [86]	3 T/NR	R2^a^ and QSM	Early-stage PD: 14/HC: 12	• ↑ PUT R2^a^ values in PD vs. HC, but no significant difference in QSM values. • ↑ Iron content in extra-basal ganglia system → correlated with non-motor symptoms, particularly sleep problems and dysautonomia, even in early-stage PD. • ↑ Iron levels in PUT of early-stage, medication-free PD.
Guan et al. (2021) [87]	3 T/8	ESWAN and QSM	PD-LC: 34/PD with normal serum ceruloplasmin: 28/HC: 121	• ↑ Significant iron accumulation in PUT in PD-LC compared to HC. • ↑ Iron accumulation in PUT remained significantly different between PD-NC and PD-LC, with negative correlation with serum ceruloplasmin in all PD. • When PD have reduced serum ceruloplasmin, more widespread iron accumulation is expected, including PUT.
Tan et al. (2021) [88]	3 T/32	QSM	PD: 47/HC: 16	• ↑ χ value significantly in PD in PUT. • ↑ MD in PD in lateral SN, PUT, and caudate, regions with the lowest χ value. • QSM and DKI complement each other, enhancing understanding of iron deposition and microstructural changes in PD pathophysiology.
Uchida et al. (2020) [89]	3 T/32	QSM	PD: 41/HC: 20	• ↑ QSM values in PUT → ↑ correlation with UPDRS-III • Striatal dopamine transporter-specific binding ratios ↔ not correlated with QSM values in SN but inversely correlated with those in striatum (PUT and caudate nucleus).
Thomas et al. (2020) [90]	3 T/64	QSM	PD: 100/HC: 37	• ↑ χ in right PUT in PD. • ↑ χ in right PUT → ↑ correlation with UPDRSIII. • ↑ QSM in PUT in PD vs. HC, indicating higher brain tissue iron content.
Uchida et al. (2019) [91]	3 T/32	QSM	PD-MCI: 24/PD: 22/HC: 20	• ↑ χ in PUT in patients with PD compared to HC group. • ↑ χ in PUT positively correlated with UPDRS-III scores in PD.
Chen et al. (2019) [57]	3 T/8	QSM	PD: 33/HC: 26	• PD group exhibited ↑ χ values in the PUT compared to HC.
Sjöström et al. (2019) [92]	3 T/20	SWI	PD: 134/PSP: 11/MSA: 10/HC: 44	• In PSP, PUT apparent χ ↑ compared to PD and HC. • In MSA, putaminal χ ↑ compared to PD and HC.
Mazzucchi et al. (2019) [93]	3 T/8	QSM	PD: 35/MSA: 12/PSP:13	• Highest χ values observed in PUT in MSA. • χ in PUT higher in both PSP and MSA compared to PD.
Xuan et al. (2017) [66]	3 T/NR	QSM	EOPD: 35/younger HC: 24 M-LOPD: 33/older HC: 22	• ↑ χ in PUT in M-LOPD group. • ↑ χ in PUT positively correlated with disease severity (bH&Y stages, UPDRS II scores, and UPDRS III scores) in M-LOPD patients, but not in EOPD.
Sjöström et al. (2017) [94]	1. 5 T and 3 T/NR	QSM	PD: 62/PSP: 15/MSA: 11/HC: 14	• PSP had the highest χ in PUT compared to all groups. • MSA exhibited ↑ χ in PUT compared to both PD and HC.
Ito et al. (2017) [95]	3 T/8	QSM	PD: 26/MSA-P: 6/MSA-C: 7/PSP: 14/HC: 20	• ↑ χ in posterior PUT in MSA-P and in anterior PUT in PSP compared to PD. • ROC analysis showed high accuracy of posterior PUT χ in differentiating PD from MSA-P groups.
Wang et al. (2017) [96]	3 T/32	SWI	MSA: 39/18 iPD/HC: 31	• ‘Swallow-tail’ sign + putaminal hypointensity ↑ accuracy in distinguishing between MSA and iPD.
Kwon et al. (2016) [97]	3 T/NR	SWI	Parkinsonian movement disorder: 62/HC: 16	• ↑ Putaminal phase value in Parkinsonian syndrome compared to HC.
Schneider et al. (2016) [98]	3 T/12	SWI	PD: 21/PIGD: 19/HC: 20	• Multivariate analysis: Lower SWI hypointensity in PUT in PIGD vs. PD patients, with a similar trend in other basal ganglia nuclei. • Pearson correlation analysis: ↑ SWI putaminal hypointensity → ↑ Tinetti total score. • SWI putaminal hypointensity potentially valuable as an imaging marker for assessing clinical progression in Parkinsonian disorders.
Azuma et al. (2016) [99]	3 T/12	QSM	PD: 24/HC: 24	• ↓ χ in PUT in PD.
Hwang et al. (2015) [100]	3 T/32	SWI	MSA-P: 27/HC: 50	• MSA-P exhibit putaminal atrophy and marked signal hypointensity.
He et al. (2015) [101]	3 T/8	QSM and T2^a^	PD: 44/HC: 35	• ↓ χ in PUT in PD.
Meijer et al. (2015) [102]	3 T/NR	SWI	PD: 38/AP: 18/MSA-P: 12/HC: 13	• ↓ Mean SWI signal intensity in PUT for MSA-P vs. PD and HC. • Severe dorsal putaminal hypointensity presence ↑ brain MR imaging accuracy. • SWI ↑ diagnostic accuracy of 3T brain MR imaging in parkinsonism by identifying severe putaminal hypointensity as indicative of multiple system atrophy-parkinsonian form. • SWI
Yoon et al. (2015) [103]	3 T/NR	SWI	PD: 30/MSA-P: 17/HC: 13	• Low signal intensity in posterior PUT may differentiate MSA-P from PD.
Wu et al. (2014) [104]	3 T/8	SWI	PD: 54 [18 patients with the Hoehn-Yahr scale <1.5 and 36 patients with the Hoehn-Yahr stage >1.5]/HC: 40	• Patients with early and intermediate/advanced PD had significantly different PUT phase values compared to HC. • PUT phase values exhibited weaker correlations with H&Y scale
Wang et al. (2012) [105]	1.5 T/8	SWI	iPD: 16/MSA-P: 8/HC: 44	• ↑ Iron deposition in PUT in patients with MSA-P compared to iPD.
Gupta et al. (2010) [106]	1.5 T/12	SWI	PD: 11/PSP: 12/MSA-P: 12/HC: 11	• ↑ Putaminal hypointensity score in PSP compared to PD.
Grabner et al. (2010) [107]	3 T/NR	SWI	PD: 25/HC: 5	• Phase increases from anterior to posterior PUT.
von Lewinski et al. (2007) [40]	1 T/NR	T2^a^	MSA: 52/PD: 88/HC: 29	• Typical T2^a^-w finding in MSA patients: Signal loss in dorsolateral PUT.

***Symbols mean: ↑: “increase” or “higher”; ↓: “decrease” or “reduced”; ← or →: “leads to” or “results in”; ↔️: “bidirectional”, “correlation”, “contribution”, “associated with”, “two-way”, and “correlate to or with".

a Abbreviations: χ (magnetic susceptibility), atypical Parkinsonian (AP), cerebellar MSA (MSA-c), diffusion kurtosis imaging (DKI), early-onset PD (EOPD), Enhanced susceptibility-weighted angiography (ESWAN), functional MRI (fMRI), globus pallidus (GP), healthy control (HC), idiopathic Parkinson's disease (iPD), magnetic resonance imaging (MRI), mean diffusivity (MD), middle-late-onset PD (M-LOPD), Montreal Cognitive Assessment (MoCA), Parkinsonian variant of multiple system atrophy (MSA-P), PD patients with mild cognitive impairment (PD-MCI), PD with normal cognition (PD-NC), PD patients with low serum ceruloplasmin (PD-LC), PD patients with normal serum ceruloplasmin (PD-NC), Postural instability and gait disorder (PIGD), predominant cerebellar ataxia (MSA-C), progressive supranuclear palsy (PSP), putamen (PUT), quantitative susceptibility mapping (QSM), receiver operating characteristic (ROC), red nucleus (RN), substantia nigra (SN), susceptibility weighted imaging (SWI), susceptibility-weighted angiography (SWAN), T2*-weighted (T2*-w), Unified Parkinson's Disease Rating Scale Part III (UPDRS-III), X-linked dystonia-parkinsonism (XDP).

b H&Y stages refer to the Hoehn and Yahr scale, which is a system used to measure the progression of Parkinson's disease.

### 3.2 Main results

We analyzed 34 studies with publication dates ranging from January 2007 to October 2023. The findings from these studies, which utilized χ MRI techniques, were diverse and provided valuable insights into the role of iron in NDDs.

#### 3.2.1 Iron accumulation patterns

According to Hanssen et al. (2023), patients with X-linked dystonia-parkinsonism (XDP) exhibit a higher χ in both the external pallidum regions and the medial PUT compared to HCs. Additionally, the authors noted iron accumulation in the anteromedial PUT, indicating that the deterioration process was initiated in these areas [77].

In patients with the Parkinsonian variant of multiple system atrophy (MSA-P), Pang et al. (2022) found that iron accumulation in the dorsolateral PUT was more intricate than in those with idiopathic PD (iPD) [78]. Additionally, Wang (2012) demonstrated that patients with MSA-P had more iron deposition in the PUT than iPD patients [105].

Several studies have investigated differences in putaminal susceptibility levels among individuals with PD, PSP, MSA, and HCs. According to Sjöström et al. (2019, 2017) [92,94], patients with PSP and MSA have higher levels of putaminal χ than those with PD and HC. In addition, Ito et al. (2017) found that the anterior PUT in PSP and posterior PUT in MSA-P showed an increase in χ compared with PD [95]. Yoon et al. (2015) [103] and Meijer et al. (2015) [102] found that the mean SWI signal intensity in the PUT for MSA-P decreased compared to PD and HC. Furthermore, Schneider et al. (2016) noted that individuals with Parkinsonism syndromes with postural instability and gait dysfunction (PIGD) had lower SWI hypointensity in the PUT than PD patients, and this putaminal hypointensity was linked to higher Tinetti total scores, suggesting its potential as an imaging marker for assessing clinical progression in these disorders [98].

#### 3.2.2 Diagnostic accuracy and efficiency

Several studies have been conducted on the efficiency of detection. For instance, Wang et al. (2017) study on the ‘Swallow-tail’ sign and putaminal hypointensity improved accuracy in distinguishing between MSA and iPD [96]. Additionally, Ito et al. (2017) [95] and Meijer et al. (2015) [102] receiver operating characteristic (ROC) analysis showed a high accuracy of posterior PUT χ in differentiating PD from MSA-P groups. Mazzucchi et al.'s two studies using QSM and morphometric imaging in 2019 and 2022 demonstrated that an increased χ of the PUT was effective in distinguishing between PD, MSA, and MSA-P, with good diagnostic accuracy [79,93]. They also found that this method was successful in differentiating PD from PSP despite some overlap between the two groups.

In a study conducted by Kang et al. (2022), the signal intensity of the QSM in the PUT was higher in patients with PD than in HC. The researchers also observed a stronger relationship between radiomic features in the PUT and Montreal Cognitive Assessment (MoCA) scores in patients with PD, indicating that it could be useful in assessing cognitive impairment in PD. Furthermore, analysis of radiomic data showed significant differences in the PUT between PD patients with mild cognitive impairment (PD-MCI), PD patients with normal cognition (PD-NC), and HC individuals [80].

#### 3.2.3 Associations with disease severity and clinical markers

Prasuhn et al. (2022) [83] and Zhao et al. (2022) [84] observed an increase in iron deposition of iPD patients in PUT and globus pallidus (GP) and an increase in χ in the bilateral PUT of PD-MCI patients, respectively. These changes are linked to high-energy-containing phosphorus metabolites in specific regions of the brain [83], as well as the severity of motor deficits [84]. Furthermore, there was a correlation between these changes and decreased MoCA-B scores and increased Unified Parkinson's Disease Rating Scale Part III (UPDRS-III) scores [84].

Several studies have reported changes in the distribution of χ in the PUT region of the brain under various conditions. These studies reported alterations in χ distribution in the PUT for MSA-P, increased PUT R2* values in PD patients compared to HCs [85], significant iron accumulation in PUT in PD patients with low serum ceruloplasmin (PD-LC) compared to HCs [87], and significantly increased χ value in PD patients with PUT [88]. Furthermore, Guan et al. (2021) [87] found that increased iron accumulation in the PUT remained significantly different between PD patients with normal serum ceruloplasmin and PD-LC, and this accumulation was negatively correlated with serum ceruloplasmin in all patients with PD. Therefore, when patients with PD have reduced serum ceruloplasmin, more widespread iron accumulation is expected, including in the PUT region of the brain.

Several studies have reported that patients with PD have higher χ levels in PUT than in HC [57,66,95,96,[89], [90], [91]]. Moreover, the increase in χ levels was found to be associated with higher UPDRS-III scores in patients with PD, indicating elevated levels of brain tissue iron content.

Increased levels of iron in the PUT are associated with more severe symptoms, cognitive decline, and motor deficits in PD patients [66,84,91,90]. One study has shown that even in the early stages of PD, higher iron content in the extra-basal ganglia system is linked to non-motor symptoms, such as sleep problems and dysautonomia [86]. Additionally, a positive correlation was found between increased χ in the PUT and disease severity (as measured by the Hoehn and Yahr scale (H&Y scale), UPDRS II scores, and UPDRS III scores) in M-LOPD patients, but not in EOPD [66]. However, some studies have reported inconsistencies, with a few finding no differences in iron deposition between PD patients and HCs [86,82]. Furthermore, although striatal dopamine transporter-specific binding ratios are not correlated with QSM values in the SN, they are inversely correlated with those in the striatum (PUT and caudate nucleus) [89].

Several studies have evaluated phase alterations and found that in Parkinsonism syndrome, there is an increase in the putaminal phase value compared with HCs [97]. Patients with early and intermediate/advanced PD also showed significantly different putaminal phase values compared with HCs [104]. Additionally, weaker correlations were observed between putaminal phase values and the H&Y scale [104], and there was an increase in the phase from anterior to posterior PUT [107].

#### 3.2.4 Associations with other neuroimaging findings

In 2022, Zang et al. discovered a noteworthy correlation between the deposition of iron in the nigral region and connectivity between the nigral and PUT regions [81]. However, in the same year, Du et al. did not observe any variation in iron levels in the PUT region between patients with PD who had not received medication and HCs [82]. Additionally, there was an increase in χ in individuals with PD-MCI, but no significant difference was observed between the PD-NC and HCs [84].

One study found an increase in mean diffusivity (MD) in certain areas of the brain, such as the lateral SN, PUT, and caudate regions, with the lowest χ value in patients with PD [88]. The use of both QSM and diffusion kurtosis imaging (DKI) provided a more comprehensive understanding of the changes in iron deposition and microstructure in PD pathophysiology [88].

A few studies have found significant differences in the χ of PUT in patients with PD, with decreased susceptibility observed in some cases [99,101]. In contrast, patients with MSA-P show signs of increased putaminal atrophy and marked signal hypointensity [100]. Another study found that using 3T brain MR can increase diagnostic accuracy in Parkinsonism by identifying severe putaminal hypointensity as an indicator of MSA-P [102]. Furthermore, patients with PSP had a higher putaminal hypointensity score than PD patients, and MSA patients showed a typical T2*-w finding, with signal loss in the dorsolateral PUT [102].

## 4 Discussion

Iron deposition in the PUT has been implicated in the pathogenesis of PD and other Parkinsonian syndromes. Magnetic susceptibility MRI techniques provide a noninvasive method for assessing iron deposition in the brain. These findings indicate that magnetic susceptibility mapping techniques such as QSM, SWI, and phase imaging can detect differences in iron accumulation in the PUT between PD, atypical Parkinsonian disorders, and HCs. Additionally, alterations in putaminal susceptibility values appear to correlate with disease severity, motor deficits, and cognitive decline.

The majority of studies included in this review found evidence of increased iron deposition in the PUT in these patients [40,57,66,[77], [78], [79], [80], [83], [84], [85], [86], [87], [88], [89], [90], [91], [92], [93], [94], [95], [96], [98], [102], [103], [105],^97^,^107^,^100^,^106^]. This finding is consistent with previous studies that have shown increased iron levels in the PUT of other NDDs [10,108,109]. The increased iron deposition in the PUT in PD is thought to be due to a combination of factors, including decreased iron transport from the brain, increased iron uptake into the brain, and increased iron storage in the brain [59,72].

Across multiple studies, increased magnetic susceptibility, as measured by QSM, was consistently found in the PUT of PD patients compared to HCs [57,80,87,[89], [90], [91]], suggesting greater iron deposition. This supports the notion that iron accumulation occurs within the basal ganglia, especially the PUT, as a part of the neurodegenerative process [10,70]. Higher QSM values were positively correlated with worse motor scores on the UPDRS-III [66,84,[89], [90], [91]], indicating an association between putaminal iron accumulation and severity of motor symptoms.

Recent investigations have employed radiomic analysis of QSM to examine iron deposition in the PUT and its association with Parkinsonian syndromes [80,84]. Notably, Kang et al. (2022) discovered that increased radiomic features derived from PUT were linked to lower MoCA scores in patients with PD, suggesting that these features could assist in evaluating cognitive impairment [80]. Similarly, Zhao et al. (2022) utilized radiomic analysis to identify significant variations in bilateral PUT among PD-MCI, PD patients with normal cognition, and HCs [84]. Moreover, elevated radiomic features in the PUT have been found to correlate with more severe motor deficits [84] and cognitive decline [80,84] in PD. Although larger studies are required, preliminary findings suggest that radiomic analysis of QSM-MRI data has the potential to quantify subtle alterations in PUT iron deposition and establish connections with PD progression and symptoms [10,110]. Radiomics may emerge as a valuable imaging biomarker for monitoring iron accumulation, motor dysfunction, and cognitive decline in PD and Parkinsonism disorders [80,84,111]. Importantly, Zhao et al. (2022) found that increased bilateral putaminal susceptibility differentiated PD patients with MCI from those with normal cognition and HCs [84]. Susceptibility changes were also negatively correlated with MoCA-B scores, linking putaminal iron deposition to cognitive decline in PD. This concurs with Kang et al. (2022), who discovered stronger correlations between putaminal radiomic features and MoCA scores in PD patients than in HCs [80]. Together, these findings imply that iron accumulation in the PUT may contribute to the pathophysiology underlying the motor and non-motor symptoms in PD. This indicates QSM's usefulness of QSM in monitoring PD progression and correlating imaging biomarkers with clinical features.

In Parkinsonism syndromes such as PSP and MSA, evidence for differences in putaminal susceptibility compared to PD is more variable [40,[78], [92], [94], [95], [102], [103], [105],[79], [93], [96],^85^,^97^,^100^,^106^]. Sjöström et al. (2017, 2019) reported higher susceptibility values in the PUT for PSP and MSA than for PD and HCs using QSM [92,94]. Similarly, Ito et al. (2017) found increased susceptibility posteriorly for MSA-P and anteriorly for PSP versus PD [95]. Moreover, other SWI studies noted reduced signal intensity and hypointensity in the PUT for MSA compared with PD [103,102]. Therefore, alterations in putaminal susceptibility distinguish PSP and MSA from PD with good diagnostic accuracy in several studies, highlighting their utility as MRI biomarkers [95,102,96,79]. Furthermore, compared to PD, PSP and MSA show more marked functional impairments, atrophy, and neurodegeneration, which could drive greater iron accumulation [40,[78], [92], [94], [95], [105],^79^,^93^,^85^].

Some studies have used SWI to assess putaminal changes in Parkinsonism [[77], [78], [92], [105],[96], [98], [102], [103],^83^,[81], [97], [104], [107],^100^,^106^]. Wu et al. (2014) demonstrated reduced SWI phase values in the PUT of PD patients compared to HCs [104]. Schneider et al. (2016) associated lower SWI hypointensity in the PUT with greater postural instability and gait difficulty in PD [98]. SWI combines both magnitude and phase information to visually represent variations in the magnetic field of tissues. In contrast, QSM quantifies the magnetic susceptibilities of these field variations [13,45]. While SWI contrast reflects various factors such as iron, myelin, calcium, and vessel architecture, combining SWI with QSM can offer complementary information [13,44,112]. Furthermore, the quantitative nature of QSM enables the longitudinal monitoring of disease progression, comparison across subjects, and potentially even between different imaging centers while minimizing observer bias [14,45,113]. However, QSM requires a series of advanced post-processing steps that rely on understanding the relationship between the magnetic susceptibility, magnetic field, and MR signal [15,114,115].

Notably, the associations between putaminal susceptibility measures and disease severity are more consistent in PD than in atypical Parkinsonism [66,84]. While higher QSM values correlated with UPDRS-III scores in PD [84,[89], [90], [91]], Xuan et al. (2017) only found this relationship for middle-late-onset PD, but not early onset PD [66]. This suggests that factors other than iron may contribute substantially to the progression of atypical Parkinsonism. Alternatively, the more advanced neurodegeneration in PSP and MSA could obscure the correlations observed in early stage PD.

Our review consistently demonstrated the presence of iron accumulation in various subregions of the PUT in Parkinsonian disorders. These specific subregions include the medial PUT in XDP [77], posterior and dorsal/dorsolateral PUT in MSA-P [78,[95], [102], [103]], and anterior PUT in PSP [95]. These findings indicate that these methods can assist in the early and precise diagnosis of Parkinsonian disorders, enabling timely treatment and management of different subregions of the PUT. In summary, consistent identification of iron deposition in specific subregions of the PUT offers valuable insights into the diagnosis and treatment of Parkinsonian disorders. Furthermore, the relationship between nigral and striatal iron deposition requires further research. One study found a significant interaction between nigral iron and nigrostriatal connectivity [81], whereas another reported no association between nigral QSM values and striatal dopamine transporter binding [89].

Several limitations of this study should be considered when interpreting its findings. Variations in MRI protocols, including the use of different techniques, such as QSM and SWI, as well as differences in analysis methods and post-processing pipelines, could contribute to the inconsistent results observed across studies. Additionally, the number of coil channels used during MRI acquisition may also affect the outcomes. These factors suggest that further technical refinements are necessary to improve the accuracy of these imaging techniques [116]. It is crucial to establish standardized protocols in future investigations to minimize discrepancies and improve comparability between studies, especially when using QSM [116]. Furthermore, a limited number of studies have reported correlations between imaging findings and the pathological or biochemical confirmation of iron levels. Conducting additional clinicopathological correlative studies would be valuable for validating the specificity of MRI measurements for assessing iron deposition.

Despite the limitations of the studies included in this review, the findings suggest that SWI/QSM techniques can be used to detect iron deposition in the PUT in PD and other Parkinsonian syndromes. Current evidence indicates that increased putaminal iron deposition occurs in PD and, to a greater extent, in atypical Parkinsonism disorders such as MSA and PSP. Thus, future studies should compare the accumulation of iron because of the limited knowledge of iron deposition differences between PSP and MSA. The role of iron accumulation as a potential diagnostic and progression imaging biomarker shows promise in establishing its clinical utility. Advanced magnetic susceptibility MRI techniques could be used to identify patients with PD who are at risk of developing dementia, such as frontotemporal dementia with parkinsonism. Multimodal advanced MRI combined with susceptibility mapping with other modalities may provide further insights into the relationship between iron dysregulation, neurodegeneration, and clinical deficits in Parkinsonism. Additionally, SWI/QSM can be used to monitor the response to iron chelation therapy in PD and other Parkinsonian syndromes.

## 5 Conclusion

Magnetic susceptibility MRI techniques can quantitatively detect putaminal iron deposition in Parkinsonian syndromes. Evidence suggests that increased putaminal susceptibility in PD correlates with greater disease severity and cognitive decline. Comparisons between PD and atypical Parkinsonian disorders show both similarities and differences in putaminal iron accumulation, which may aid in the differential diagnosis. Current evidence suggests that there is more iron deposition in the PUT in atypical Parkinsonism disorders such as MSA and PSP compared to PD. Going forward, larger multi-site studies with correlative pathology and longitudinal multimodal imaging will help further define the role of aberrant iron homeostasis in PUT and its relationship with clinical progression in PD and related disorders. This emerging body of work highlights the potential diagnostic and prognostic utility of advanced iron-sensitive MRI methods as biomarkers for PD and atypical Parkinsonian syndromes.

## Ethics approval and consent to participate

Not applicable.

## Consent for publication

Not applicable.

## Availability of data and materials

This article contains all of the data produced or analyzed during this investigation. Any further inquiries should be forwarded to the corresponding author.

## Funding

This research did not receive any specific grant from funding agencies in the public, commercial, or not-for-profit sectors.

## Data availability statement

Data will be made available on request.

## CRediT authorship contribution statement

**Sana Mohammadi:** Writing – review & editing, Writing – original draft, Visualization, Validation, Project administration, Methodology, Investigation, Formal analysis. **Sadegh Ghaderi:** Writing – review & editing, Writing – original draft, Visualization, Validation, Supervision, Project administration, Methodology, Investigation, Formal analysis, Data curation, Conceptualization.

## Declaration of competing interest

The authors declare that they have no known competing financial interests or personal relationships that could have appeared to influence the work reported in this paper.

## References

[1] Ghaderi S., Mohammadi S., Nezhad N.J., Karami S., Sayehmiri F. (2024). Iron quantification in basal ganglia: quantitative susceptibility mapping as a potential biomarker for Alzheimer's disease – a systematic review and meta-analysis. Front. Neurosci..

[2] Mohammadi S., Mohammadi M., Ghaderi S. (2023). Sleep-related regions in neurodegenerative diseases by central nervous system localization using magnetic resonance imaging. Psychiatr. Res. Neuroimaging.

[3] Ghaderi S., Batouli S.A.H., Mohammadi S., Fatehi F. (2023). Iron quantification in basal ganglia using quantitative susceptibility mapping in a patient with ALS: a case report and literature review. Front. Neurosci..

[4] Ghaderi S., Mohammadi S. (2023). Motor band sign or biomarker. iRADIOLOGY.

[5] Duyn J. (2013). MR susceptibility imaging. J. Magn. Reson..

[6] Duyn J.H., Schenck J. (2017). Contributions to magnetic susceptibility OF brain tissue. NMR Biomed..

[7] Klohs J., Hirt A.M. (2021). Investigation of the magnetic susceptibility properties of fresh and fixed mouse heart, liver, skeletal muscle and brain tissue. Phys. Med..

[8] Abbaspour N., Hurrell R., Kelishadi R. (2014). Review on iron and its importance for human health. J. Res. Med. Sci..

[9] Cronin S.J.F., Woolf C.J., Weiss G., Penninger J.M. (2019). The role of iron regulation in immunometabolism and immune-related disease. Front. Mol. Biosci..

[10] Ravanfar P., Loi S.M., Syeda W.T., Van Rheenen T.E., Bush A.I., Desmond P., Cropley V.L., Lane D.J.R., Opazo C.M., Moffat B.A., Velakoulis D., Pantelis C. (2021). Systematic review: quantitative susceptibility mapping (QSM) of brain iron profile in neurodegenerative diseases. Front. Neurosci..

[11] Yan S.-Q., Sun J.-Z., Yan Y.-Q., Wang H., Lou M. (2012). Evaluation of brain iron content based on magnetic resonance imaging (MRI): comparison among phase value, R2* and magnitude signal intensity. PLoS One.

[12] Tambasco N., Nigro P., Chiappiniello A., Paolini Paoletti F., Scialpi S., Simoni S., Chiarini P., Parnetti L. (2022). An updated overview of the magnetic resonance imaging of brain iron in movement disorders. Behav. Neurol..

[13] Liu C., Li W., Tong K.A., Yeom K.W., Kuzminski S. (2015). Susceptibility-weighted imaging and quantitative susceptibility mapping in the brain. J. Magn. Reson. Imag..

[14] Wang Y., Spincemaille P., Liu Z., Dimov A., Deh K., Li J., Zhang Y., Yao Y., Gillen K.M., Wilman A.H., Gupta A., Tsiouris A.J., Kovanlikaya I., Chiang G.C.-Y., Weinsaft J.W., Tanenbaum L., Chen W., Zhu W., Chang S., Lou M., Kopell B.H., Kaplitt M.G., Devos D., Hirai T., Huang X., Korogi Y., Shtilbans A., Jahng G.-H., Pelletier D., Gauthier S.A., Pitt D., Bush A.I., Brittenham G.M., Prince M.R. (2017). Clinical quantitative susceptibility mapping (QSM) – biometal imaging and its emerging roles in patient care. J. Magn. Reson. Imag..

[15] Li Z., Feng R., Liu Q., Feng J., Lao G., Zhang M., Li J., Zhang Y., Wei H. (2023). APART-QSM: an improved sub-voxel quantitative susceptibility mapping for susceptibility source separation using an iterative data fitting method. Neuroimage.

[16] Schreiner O.D., Schreiner T.G. (2023). Iron chelators as a therapeutic option for Alzheimer's disease—a mini-review. Front. Aging.

[17] Reeves J.A., Mohebbi M., Zivadinov R., Bergsland N., Dwyer M.G., Salman F., Schweser F., Jakimovski D. (2023). Reliability of paramagnetic rim lesion classification on quantitative susceptibility mapping (QSM) in people with multiple sclerosis: single-site experience and systematic review. Mult Scler Relat Disord.

[18] Wang Y., He N., Zhang C., Zhang Y., Wang C., Huang P., Jin Z., Li Y., Cheng Z., Liu Y., Wang X., Chen C., Cheng J., Liu F., Haacke E.M., Chen S., Yang G., Yan F. (2023). An automatic interpretable deep learning pipeline for accurate Parkinson's disease diagnosis using quantitative susceptibility mapping and T1-weighted images. Hum. Brain Mapp..

[19] Shibata H., Uchida Y., Inui S., Kan H., Sakurai K., Oishi N., Ueki Y., Oishi K., Matsukawa N. (2022). Machine learning trained with quantitative susceptibility mapping to detect mild cognitive impairment in Parkinson's disease. Parkinsonism Relat. Disorders.

[20] Ahmed M., Chen J., Arani A., Senjem M.L., Cogswell P.M., Jack C.R., Liu C. (2023). The diamagnetic component map from quantitative susceptibility mapping (QSM) source separation reveals pathological alteration in Alzheimer's disease-driven neurodegeneration. Neuroimage.

[21] Uchida Y., Kan H., Sakurai K., Oishi K., Matsukawa N. (2022). Quantitative susceptibility mapping as an imaging biomarker for Alzheimer's disease: the expectations and limitations. Front. Neurosci..

[22] Kan H., Uchida Y., Arai N., Ueki Y., Aoki T., Kasai H., Kunitomo H., Hirose Y., Matsukawa N., Shibamoto Y. (2020). Simultaneous voxel-based magnetic susceptibility and morphometry analysis using magnetization-prepared spoiled turbo multiple gradient echo. NMR Biomed..

[23] Yao J., Morrison M.A., Jakary A., Avadiappan S., Chen Y., Luitjens J., Glueck J., Driscoll T., Geschwind M.D., Nelson A.B., Villanueva-Meyer J.E., Hess C.P., Lupo J.M. (2023). Comparison of quantitative susceptibility mapping methods for iron-sensitive susceptibility imaging at 7T: an evaluation in healthy subjects and patients with Huntington's disease. Neuroimage.

[24] Mohammadi S., Ghaderi S. (2023). Motor band sign in motor neuron diseases using magnetic resonance imaging: a systematic review. Acta Neurol. Scand..

[25] Zhou X.-X., Qin H.-L., Li X.-H., Huang H.-W., Liang Y.-Y., Liang X.-L., Pu X.-Y. (2014). Characterizing brain mineral deposition in patients with Wilson disease using susceptibility-weighted imaging. Neurol. India.

[26] Shrimanker I., Tadi P., Sánchez-Manso J.C. (2023). http://www.ncbi.nlm.nih.gov/books/NBK542224/.

[27] DeMaagd G., Philip A. (2015). Parkinson's disease and its management. P T.

[28] Magrinelli F., Picelli A., Tocco P., Federico A., Roncari L., Smania N., Zanette G., Tamburin S. (2016). Pathophysiology of motor dysfunction in Parkinson's disease as the rationale for drug treatment and rehabilitation. Parkinson's Dis..

[29] Greenland J.C., Barker R.A., Stoker T.B., Greenland J.C. (2018). Parkinson's Disease: Pathogenesis and Clinical Aspects.

[30] Dickson D.W. (2012). Parkinson's disease and parkinsonism: neuropathology. Cold Spring Harb Perspect Med.

[31] Kouli A., Torsney K.M., Kuan W.-L., Stoker T.B., Greenland J.C. (2018). Parkinson's Disease: Pathogenesis and Clinical Aspects.

[32] Brooks D.J. (2012). Parkinson's disease: diagnosis. Parkinsonism Relat. Disorders.

[33] Bunzeck N., Singh-Curry V., Eckart C., Weiskopf N., Perry R.J., Bain P.G., Düzel E., Husain M. (2013). Motor phenotype and magnetic resonance measures of basal ganglia iron levels in Parkinson's disease. Parkinsonism Relat. Disorders.

[34] Devignes Q., Lopes R., Dujardin K. (2022). Neuroimaging outcomes associated with mild cognitive impairment subtypes in Parkinson's disease: a systematic review. Park. Relat. Disord..

[35] McFarland N.R. (2016). Diagnostic approach to atypical parkinsonian syndromes. Continuum.

[36] Ananthavarathan P., Patel B., Peeros S., Obrocki R., Malek N. (2023). Neurological update: non-motor symptoms in atypical parkinsonian syndromes. J. Neurol..

[37] Ryman S.G., Poston K.L. (2020). MRI biomarkers of motor and non-motor symptoms in Parkinson's disease. Park. Relat. Disord..

[38] Shin H.-W., Hong S.-W., Youn Y.C. (2022). Clinical aspects of the differential diagnosis of Parkinson's disease and parkinsonism. J. Clin. Neurol..

[39] Jurgens C.K., Jasinschi R., Ekin A., Witjes-Ané M.-N.W., van der Grond J., Middelkoop H., Roos R.A.C. (2010). MRI T2 Hypointensities in basal ganglia of premanifest Huntington's disease. PLoS Curr.

[40] von Lewinski F., Werner C., Jörn T., Mohr A., Sixel-Döring F., Trenkwalder C. (2007). T2*-weighted MRI in diagnosis of multiple system atrophy. A practical approach for clinicians. J. Neurol..

[41] Yang J., Li X., Yang R., Yu X., Yu C., Qian Y., Yu Y. (2015). Susceptibility-weighted imaging manifestations in the brain of wilson's disease patients. PLoS One.

[42] Alexopoulou E., Stripeli F., Baras P., Seimenis I., Kattamis A., Ladis V., Efstathopoulos E., Brountzos E.N., Kelekis A.D., Kelekis N.L. (2006). R2 relaxometry with MRI for the quantification of tissue iron overload in beta-thalassemic patients. J. Magn. Reson. Imag..

[43] Haacke E.M., Mittal S., Wu Z., Neelavalli J., Cheng Y.-C.N. (2009). Susceptibility-weighted imaging: technical aspects and clinical applications, part 1. AJNR Am J Neuroradiol.

[44] Haller S., Haacke E.M., Thurnher M.M., Barkhof F. (2021). Susceptibility-weighted imaging: technical essentials and clinical neurologic applications. Radiology.

[45] Ruetten P.P.R., Gillard J.H., Graves M.J. (2019). Introduction to quantitative susceptibility mapping and susceptibility weighted imaging. Br. J. Radiol..

[46] Boss A., Heeb L., Vats D., Starsich F.H.L., Balfourier A., Herrmann I.K., Gupta A. (2022). Assessment of iron nanoparticle distribution in mouse models using ultrashort‐echo‐time MRI. NMR Biomed..

[47] Sigirli D., Ozdemir S.T., Erer S., Sahin I., Ercan I., Ozpar R., Orun M.O., Hakyemez B. (2021). Statistical shape analysis of putamen in early-onset Parkinson's disease. Clin. Neurol. Neurosurg..

[48] Luo X., Mao Q., Shi J., Wang X., Li C.-S.R. (2019). Putamen gray matter volumes in neuropsychiatric and neurodegenerative disorders. World J Psychiatry Ment Health Res.

[49] de Jong L.W., van der Hiele K., Veer I.M., Houwing J.J., Westendorp R.G.J., Bollen E.L.E.M., de Bruin P.W., Middelkoop H.A.M., van Buchem M.A., van der Grond J. (2008). Strongly reduced volumes of putamen and thalamus in Alzheimer's disease: an MRI study. Brain.

[50] Pievani M., Bocchetta M., Boccardi M., Cavedo E., Bonetti M., Thompson P.M., Frisoni G.B. (2013). Striatal morphology in early-onset and late-onset Alzheimer's disease: a preliminary study. Neurobiol. Aging.

[51] Machts J., Loewe K., Kaufmann J., Jakubiczka S., Abdulla S., Petri S., Dengler R., Heinze H.-J., Vielhaber S., Schoenfeld M.A., Bede P. (2015). Basal ganglia pathology in ALS is associated with neuropsychological deficits. Neurology.

[52] Ghaderi S., Fatehi F., Kalra S., Batouli S.A.H. (2023). MRI biomarkers for memory-related impairment in amyotrophic lateral sclerosis: a systematic review. Amyotrophic Lateral Sclerosis and Frontotemporal Degeneration.

[53] Ghaderi S., Mohammadi S., Mohammadi M. (2023). Obstructive sleep apnea and attention deficits: a systematic review of magnetic resonance imaging biomarkers and neuropsychological assessments. Brain Behav.

[54] Kumar R., Farahvar S., Ogren J.A., Macey P.M., Thompson P.M., Woo M.A., Yan-Go F.L., Harper R.M. (2014). Brain putamen volume changes in newly-diagnosed patients with obstructive sleep apnea. Neuroimage: Clinic.

[55] Sacchet M.D., Camacho M.C., Livermore E.E., Thomas E.A.C., Gotlib I.H. (2017). Accelerated aging of the putamen in patients with major depressive disorder. J. Psychiatr. Neurosci..

[56] Lee J.-H., Lee M.-S. (2019). Brain iron accumulation in atypical parkinsonian syndromes: in vivo MRI evidences for distinctive patterns. Front. Neurol..

[57] Chen Q., Chen Y., Zhang Y., Wang F., Yu H., Zhang C., Jiang Z., Luo W. (2019). Iron deposition in Parkinson's disease by quantitative susceptibility mapping. BMC Neurosci..

[58] Berg D., Hochstrasser H. (2006). Iron metabolism in Parkinsonian syndromes. Mov. Disord..

[59] Foley P.B., Hare D.J., Double K.L. (2022). A brief history of brain iron accumulation in Parkinson disease and related disorders. J. Neural. Transm..

[60] Fioravanti V., Benuzzi F., Codeluppi L., Contardi S., Cavallieri F., Nichelli P., Valzania F. (2015). MRI correlates of Parkinson's disease progression: a voxel based morphometry study. Parkinson's Dis..

[61] Foffani G., Obeso J.A. (2018). A cortical pathogenic theory of Parkinson's disease. Neuron.

[62] Weingarten C.P., Sundman M.H., Hickey P., Chen N. (2015). Neuroimaging of Parkinson's disease: expanding views. Neurosci. Biobehav. Rev..

[63] Kordower J.H., Olanow C.W., Dodiya H.B., Chu Y., Beach T.G., Adler C.H., Halliday G.M., Bartus R.T. (2013). Disease duration and the integrity of the nigrostriatal system in Parkinson's disease. Brain.

[64] Madden D.J., Merenstein J.L. (2023). Quantitative susceptibility mapping of brain iron in healthy aging and cognition. Neuroimage.

[65] Wang J.-Y., Zhuang Q.-Q., Zhu L.-B., Zhu H., Li T., Li R., Chen S.-F., Huang C.-P., Zhang X., Zhu J.-H. (2016). Meta-analysis of brain iron levels of Parkinson's disease patients determined by postmortem and MRI measurements. Sci. Rep..

[66] Xuan M., Guan X., Gu Q., Shen Z., Yu X., Qiu T., Luo X., Song R., Jiaerken Y., Xu X., Huang P., Luo W., Zhang M. (2017). Different iron deposition patterns in early- and middle-late-onset Parkinson's disease. Parkinsonism Relat. Disorders.

[67] Fu X., Deng W., Cui X., Zhou X., Song W., Pan M., Chi X., Xu J., Jiang Y., Wang Q., Xu Y. (2021). Time-specific pattern of iron deposition in different regions in Parkinson's disease measured by quantitative susceptibility mapping. Front. Neurol..

[68] Dexter D.T., Jenner P., Schapira A.H., Marsden C.D. (1992). Alterations in levels of iron, ferritin, and other trace metals in neurodegenerative diseases affecting the basal ganglia. The Royal Kings and Queens Parkinson's Disease Research Group. Ann. Neurol..

[69] Mao H., Dou W., Chen K., Wang X., Wang X., Guo Y., Zhang C. (2022). Evaluating iron deposition in gray matter nuclei of patients with unilateral middle cerebral artery stenosis using quantitative susceptibility mapping. Neuroimage Clin.

[70] Daugherty A.M., Raz N. (2016). Accumulation of iron in the putamen predicts its shrinkage in healthy older adults: a multi-occasion longitudinal study. Neuroimage.

[71] Marvel C.L., Chen L., Joyce M.R., Morgan O.P., Iannuzzelli K.G., LaConte S.M., Lisinski J.M., Rosenthal L.S., Li X. (2022). Quantitative susceptibility mapping of basal ganglia iron is associated with cognitive and motor functions that distinguish spinocerebellar ataxia type 6 and type 3. Front. Neurosci..

[72] Ward R.J., Zucca F.A., Duyn J.H., Crichton R.R., Zecca L. (2014). The role of iron in brain ageing and neurodegenerative disorders. Lancet Neurol..

[73] Ndayisaba A., Kaindlstorfer C., Wenning G.K. (2019). Iron in neurodegeneration – cause or consequence?. Front. Neurosci..

[74] Page M.J., McKenzie J.E., Bossuyt P.M., Boutron I., Hoffmann T.C., Mulrow C.D., Shamseer L., Tetzlaff J.M., Akl E.A., Brennan S.E., Chou R., Glanville J., Grimshaw J.M., Hróbjartsson A., Lalu M.M., Li T., Loder E.W., Mayo-Wilson E., McDonald S., McGuinness L.A., Stewart L.A., Thomas J., Tricco A.C., Welch V.A., Whiting P., Moher D. (2021). The PRISMA 2020 statement: an updated guideline for reporting systematic reviews. BMJ.

[75] Wells G.A., Shea B., O'Connell D., Peterson J., Welch V., Losos M., Tugwell P. (2000).

[76] Lo C.K.-L., Mertz D., Loeb M. (2014). Newcastle-Ottawa Scale: comparing reviewers' to authors' assessments. BMC Med. Res. Methodol..

[77] Hanssen H., Diesta C.C.E., Heldmann M., Dy J., Tantianpact J., Steinhardt J., Sauza R., Manalo H.T.S., Sprenger A., Reyes C.J., Tuazon R., Laabs B.-H., Domingo A., Rosales R.L., Klein C., Münte T.F., Westenberger A., Oropilla J.Q., Brüggemann N. (2023). Basal ganglia atrophy as a marker for prodromal X-linked dystonia-parkinsonism. Ann. Neurol..

[78] Pang H., Yu Z., Yu H., Chang M., Cao J., Li Y., Guo M., Liu Y., Cao K., Fan G. (2022). Multimodal striatal neuromarkers in distinguishing parkinsonian variant of multiple system atrophy from idiopathic Parkinson's disease. CNS Neurosci. Ther..

[79] Mazzucchi S., Del Prete E., Costagli M., Frosini D., Paoli D., Migaleddu G., Cecchi P., Donatelli G., Morganti R., Siciliano G., Cosottini M., Ceravolo R. (2022). Morphometric imaging and quantitative susceptibility mapping as complementary tools in the diagnosis of parkinsonisms. Eur. J. Neurol..

[80] Kang J.J., Chen Y., Xu G.D., Bao S.L., Wang J., Ge M., Shen L.H., Jia Z.Z. (2022). Combining quantitative susceptibility mapping to radiomics in diagnosing Parkinson's disease and assessing cognitive impairment. Eur. Radiol..

[81] Zang Z., Song T., Li J., Yan S., Nie B., Mei S., Ma J., Yang Y., Shan B., Zhang Y., Lu J. (2022). Modulation effect of substantia nigra iron deposition and functional connectivity on putamen glucose metabolism in Parkinson's disease. Hum. Brain Mapp..

[82] Du G., Wang E., Sica C., Chen H., De Jesus S., Lewis M.M., Kong L., Connor J., Mailman R.B., Huang X. (2022). Dynamics of nigral iron accumulation in Parkinson's disease: from diagnosis to late stage. Mov. Disord..

[83] Prasuhn J., Göttlich M., Gerkan F., Kourou S., Ebeling B., Kasten M., Hanssen H., Klein C., Brüggemann N. (2022). Relationship between brain iron deposition and mitochondrial dysfunction in idiopathic Parkinson's disease. Mol. Med..

[84] Zhao Y., Qu H., Wang W., Liu J., Pan Y., Li Z., Xu G., Hu C. (2022). Assessing mild cognitive impairment in Parkinson's disease by magnetic resonance quantitative susceptibility mapping combined voxel-wise and radiomic analysis. Eur. Neurol..

[85] Lancione M., Cencini M., Costagli M., Donatelli G., Tosetti M., Giannini G., Zangaglia R., Calandra-Buonaura G., Pacchetti C., Cortelli P., Cosottini M. (2022). Diagnostic accuracy of quantitative susceptibility mapping in multiple system atrophy: the impact of echo time and the potential of histogram analysis. Neuroimage Clin.

[86] Kim M., Yoo S., Kim D., Cho J.W., Kim J.S., Ahn J.H., Mun J.K., Choi I., Lee S.-K., Youn J. (2021). Extra-basal ganglia iron content and non-motor symptoms in drug-naïve, early Parkinson's disease. Neurol. Sci..

[87] Guan X., Bai X., Zhou C., Guo T., Wu J., Gu L., Gao T., Wang X., Wei H., Zhang Y., Xuan M., Gu Q., Huang P., Liu C., Zhang B., Pu J., Song Z., Yan Y., Xu X., Zhang M. (2021). Serum ceruloplasmin depletion is associated with magnetic resonance evidence of widespread accumulation of brain iron in Parkinson's disease. J. Magn. Reson. Imag..

[88] Tan S., Hartono S., Welton T., Ann C.N., Lim S.L., Koh T.S., Li H., Setiawan F., Ng S., Chia N., Liu S., Mark Haacke E., King Tan E., Chew Seng Tan L., Ling Chan L. (2021). Utility of quantitative susceptibility mapping and diffusion kurtosis imaging in the diagnosis of early Parkinson's disease. Neuroimage Clin.

[89] Uchida Y., Kan H., Sakurai K., Inui S., Kobayashi S., Akagawa Y., Shibuya K., Ueki Y., Matsukawa N. (2020). Magnetic susceptibility associates with dopaminergic deficits and cognition in Parkinson's disease. Mov. Disord..

[90] Thomas G.E.C., Leyland L.A., Schrag A.-E., Lees A.J., Acosta-Cabronero J., Weil R.S. (2020). Brain iron deposition is linked with cognitive severity in Parkinson's disease. J. Neurol. Neurosurg. Psychiatry.

[91] Uchida Y., Kan H., Sakurai K., Arai N., Kato D., Kawashima S., Ueki Y., Matsukawa N. (2019). Voxel-based quantitative susceptibility mapping in Parkinson's disease with mild cognitive impairment. Mov. Disord..

[92] Sjöström H., Surova Y., Nilsson M., Granberg T., Westman E., van Westen D., Svenningsson P., Hansson O. (2019). Mapping of apparent susceptibility yields promising diagnostic separation of progressive supranuclear palsy from other causes of parkinsonism. Sci. Rep..

[93] Mazzucchi S., Frosini D., Costagli M., Del Prete E., Donatelli G., Cecchi P., Migaleddu G., Bonuccelli U., Ceravolo R., Cosottini M. (2019). Quantitative susceptibility mapping in atypical Parkinsonisms. Neuroimage Clin.

[94] Sjöström H., Granberg T., Westman E., Svenningsson P. (2017). Quantitative susceptibility mapping differentiates between parkinsonian disorders. Parkinsonism Relat. Disorders.

[95] Ito K., Ohtsuka C., Yoshioka K., Kameda H., Yokosawa S., Sato R., Terayama Y., Sasaki M. (2017). Differential diagnosis of parkinsonism by a combined use of diffusion kurtosis imaging and quantitative susceptibility mapping. Neuroradiology.

[96] Wang N., Yang H., Li C., Fan G., Luo X. (2017). Using “swallow-tail” sign and putaminal hypointensity as biomarkers to distinguish multiple system atrophy from idiopathic Parkinson's disease: a susceptibility-weighted imaging study. Eur. Radiol..

[97] Kwon G.H., Jang J., Choi H.S., Hwang E.-J., Jung S.-L., Ahn K.-J., Kim B.-S., Yoo I.R., Kim S.H., Haacke E.M. (2016). The phase value of putamen measured by susceptibility weighted images in Parkinson's disease and in other forms of Parkinsonism: a correlation study with F18 FP-CIT PET. Acta Radiol..

[98] Schneider E., Ng K.-M., Yeoh C.-S., Rumpel H., Fook-Chong S., Li H.-H., Tan E.-K., Chan L.-L. (2016). Susceptibility-weighted MRI of extrapyramidal brain structures in Parkinsonian disorders. Medicine (Baltim.).

[99] Azuma M., Hirai T., Yamada K., Yamashita S., Ando Y., Tateishi M., Iryo Y., Yoneda T., Kitajima M., Wang Y., Yamashita Y. (2016). Lateral asymmetry and spatial difference of iron deposition in the substantia nigra of patients with Parkinson disease measured with quantitative susceptibility mapping. AJNR Am J Neuroradiol.

[100] Hwang I., Sohn C.-H., Kang K.M., Jeon B.S., Kim H.-J., Choi S.H., Yun T.J., Kim J.-H. (2015). Differentiation of parkinsonism-predominant multiple system atrophy from idiopathic Parkinson disease using 3T susceptibility-weighted MR imaging, focusing on putaminal change and lesion asymmetry. AJNR Am J Neuroradiol.

[101] He N., Ling H., Ding B., Huang J., Zhang Y., Zhang Z., Liu C., Chen K., Yan F. (2015). Region-specific disturbed iron distribution in early idiopathic Parkinson's disease measured by quantitative susceptibility mapping. Hum. Brain Mapp..

[102] Meijer F.J.A., van Rumund A., Fasen B.A.C.M., Titulaer I., Aerts M., Esselink R., Bloem B.R., Verbeek M.M., Goraj B. (2015). Susceptibility-weighted imaging improves the diagnostic accuracy of 3T brain MRI in the work-up of parkinsonism. AJNR Am J Neuroradiol.

[103] Yoon R.G., Kim S.J., Kim H.S., Choi C.G., Kim J.S., Oh J., Chung S.J., Lee C.S. (2015). The utility of susceptibility-weighted imaging for differentiating Parkinsonism-predominant multiple system atrophy from Parkinson's disease: correlation with 18F-flurodeoxyglucose positron-emission tomography. Neurosci. Lett..

[104] Wu S.-F., Zhu Z.-F., Kong Y., Zhang H.-P., Zhou G.-Q., Jiang Q.-T., Meng X.-P. (2014). Assessment of cerebral iron content in patients with Parkinson's disease by the susceptibility-weighted MRI. Eur. Rev. Med. Pharmacol. Sci..

[105] Wang Y., Butros S.R., Shuai X., Dai Y., Chen C., Liu M., Haacke E.M., Hu J., Xu H. (2012). Different iron-deposition patterns of multiple system atrophy with predominant parkinsonism and idiopathetic Parkinson diseases demonstrated by phase-corrected susceptibility-weighted imaging. AJNR Am J Neuroradiol.

[106] Gupta D., Saini J., Kesavadas C., Sarma P.S., Kishore A. (2010). Utility of susceptibility-weighted MRI in differentiating Parkinson's disease and atypical parkinsonism. Neuroradiology.

[107] Grabner G., Haubenberger D., Rath J., Beisteiner R., Auff E., Trattnig S., Barth M. (2010). A population-specific symmetric phase model to automatically analyze susceptibility-weighted imaging (SWI) phase shifts and phase symmetry in the human brain. J. Magn. Reson. Imag..

[108] Sugiyama A., Sato N., Kimura Y., Fujii H., Maikusa N., Shigemoto Y., Suzuki F., Morimoto E., Koide K., Takahashi Y., Matsuda H., Kuwabara S. (2019). Quantifying iron deposition in the cerebellar subtype of multiple system atrophy and spinocerebellar ataxia type 6 by quantitative susceptibility mapping. J. Neurol. Sci..

[109] Eskreis-Winkler S., Zhang Y., Zhang J., Liu Z., Dimov A., Gupta A., Wang Y. (2017). The clinical utility of QSM: disease diagnosis, medical management, and surgical planning. NMR Biomed..

[110] Liu Y., Xiao B., Zhang C., Li J., Lai Y., Shi F., Shen D., Wang L., Sun B., Li Y., Jin Z., Wei H., Haacke E.M., Zhou H., Wang Q., Li D., He N., Yan F. (2021). Predicting motor outcome of subthalamic nucleus deep brain stimulation for Parkinson's disease using quantitative susceptibility mapping and radiomics: a pilot study. Front. Neurosci..

[111] Shu Z., Pang P., Wu X., Cui S., Xu Y., Zhang M. (2020). An integrative nomogram for identifying early-stage Parkinson's disease using non-motor symptoms and white matter-based radiomics biomarkers from whole-brain MRI. Front. Aging Neurosci..

[112] Halefoglu A.M., Yousem D.M. (2018). Susceptibility weighted imaging: clinical applications and future directions. World J. Radiol..

[113] Bhattarai A., Chen Z., Ward P.G.D., Talman P., Mathers S., Phan T.G., Chapman C., Howe J., Lee S., Lie Y., Egan G.F., Chua P. (2020). Serial assessment of iron in the motor cortex in limb-onset amyotrophic lateral sclerosis using quantitative susceptibility mapping. Quant. Imag. Med. Surg..

[114] Kim S., Lee Y., Jeon C.-Y., Kim K., Jeon Y., Jin Y.B., Oh S., Lee C. (2020). Quantitative magnetic susceptibility assessed by 7T magnetic resonance imaging in Alzheimer's disease caused by streptozotocin administration. Quant. Imag. Med. Surg..

[115] Aimo A., Huang L., Tyler A., Barison A., Martini N., Saccaro L.F., Roujol S., Masci P.-G. (2022). Quantitative susceptibility mapping (QSM) of the cardiovascular system: challenges and perspectives. J. Cardiovasc. Magn. Reson..

[116] Q.C.O. Committee, B. Bilgic, M. Costagli, K.-S. Chan, J. Duyn, C. Langkammer, J. Lee, X. Li, C. Liu, J.P. Marques, C. Milovic, S.D. Robinson, F. Schweser, K. Shmueli, P. Spincemaille, S. Straub, P. van Zijl, Y. Wang, I.E.-M.T.P.S. Group, Recommended implementation of quantitative susceptibility mapping for clinical research in the brain: A consensus of the ISMRM electro-magnetic tissue properties study group, Magnetic Resonance in Medicine n/a (n.d.). 10.1002/mrm.30006.. PMC10950544 38247051

