# Supplementary material for: Parkinson's disease and Parkinsonism syndromes: Evaluating iron deposition in the putamen using magnetic susceptibility MRI techniques - A systematic review and literature analysis
Source: Heliyon. 2024 Mar 26;10(7):e27950. doi: 10.1016/j.heliyon.2024.e27950 (PMC11059419; doi:10.1016/j.heliyon.2024.e27950)
Supplement: Multimedia component 1 [file mmc1.docx]

| **Supplementary Table 1.** Search results on PubMed and Scopus databases for Parkinson's disease and Parkinsonism syndrome | | | |  |
| --- | --- | --- | --- | --- |
| **Database** | **Terms** | **Search Keywords** | **Number of initial record findings** | **After removed non-English records** |
| **PubMed** | All Fields | ("Parkinsonism" OR "Parkinsonism syndrome" OR "Parkinson's disease" OR "Multiple system atrophy" OR "Progressive supranuclear palsy" OR "Lewy Body Dementia" OR "Corticobasal degeneration") AND ("Magnetic resonance imaging" OR "MRI" OR "Quantitative susceptibility Mapping" OR "QSM" OR "Susceptibility-weighted imaging" OR "SWI" OR "T2-weighted" OR "R2") AND ("Iron" OR "Iron deposition" OR "iron accumulation") AND ("Putamen") | 136 | 129 |
| **Scopus** | Title/Abstract | (TITLE-ABS-KEY("Parkinsonism") OR TITLE-ABS-KEY("Parkinsonism syndrome") OR TITLE-ABS-KEY("Parkinson's disease") OR TITLE-ABS-KEY("Multiple system atrophy") OR TITLE-ABS-KEY("Progressive supranuclear palsy") OR TITLE-ABS-KEY("Lewy Body Dementia") OR TITLE-ABS-KEY("Corticobasal degeneration")) AND (TITLE-ABS-KEY("Magnetic resonance imaging") OR TITLE-ABS-KEY(MRI) OR TITLE-ABS-KEY("Quantitative susceptibility Mapping") OR TITLE-ABS-KEY(QSM) OR TITLE-ABS-KEY("Susceptibility-weighted imaging") OR TITLE-ABS-KEY(SWI) OR TITLE-ABS-KEY("T2-weighted") OR TITLE-ABS-KEY("T2*-weighted") OR TITLE-ABS-KEY(R2) OR TITLE-ABS-KEY(R2*)) AND (TITLE-ABS-KEY(Iron) OR TITLE-ABS-KEY("Iron deposition") OR TITLE-ABS-KEY("iron accumulation")) AND (TITLE-ABS-KEY(Putamen)) | 193 | 181 |
